# Supplementary material for: Multiscale biochemical mapping of the brain through deep-learning-enhanced high-throughput mass spectrometry
Source: Nat Methods. 2024 Feb 16;21(3):521–30. doi: 10.1038/s41592-024-02171-3 (PMC10927565; doi:10.1038/s41592-024-02171-3)
Supplement: Supplementary file 1 — Supplementary Protocols and Supplementary Figs. 1–10. [file 41592_2024_2171_MOESM1_ESM.pdf]

nature methods

Article

https://doi.org/10.1038/s41592-024-02171-3

Multiscale biochemical mapping of the brain through deep-learning-enhanced high-throughput mass spectrometry

In the format provided by the authors and unedited

Table of Contents

Supplementary Protocol

Supplemental Protocol 1. Network training and reconstruction.............................S2
Supplemental Protocol 2. Deformable multimodal image registration using parametric UMAP derived feature maps.......................S7
Supplemental Protocol 3. MSI data brain regional analysis and MSI+SCMS data integration S13

Supplementary Figures

Supplementary Fig 1. Reconstructed signals on adjacent sagittal sections S17
Supplementary Fig 2. Quantitative evaluation of MEISTER S18
Supplementary Fig 3. Reconstruction of 3D MSI data with sagittal sections S19
Supplementary Fig 4. Parametric UMAP embedding of sagittal brain sections S20
Supplementary Fig 5. Individual section UMAP of lipid profiles from 3D coronal data S21
Supplementary Fig 6. Classification of brain regions through machine learning S22
Supplementary Fig 7. Interpretable machine learning on brain region prediction ...S23
Supplementary Fig 8. Differential analysis and joint fitting of single cell data S24
Supplementary Fig 9. Analysis of hippocampal single cells...................................S25
Supplementary Fig 10. Dice Index between brain atlas and human-annotated masks S26

Supplementary Protocol Summary Information

Sample preparation

Tissue sections for mass spectrometry imaging (MSI) were prepared using fresh-frozen tissue sections as described in previous work.[1] For single-cell mass spectrometry (SCMS), dissected tissue sections were dissociated into single cells with papain and spread onto indium tin oxide (ITO)-coated glass slides (Online Methods). The slides were imaged with brightfield and fluorescence microscopy. The open-source software package microMS was used for (1) object detection of single cells (2) transformation of single cell coordinates in microscopy images to mass spectrometer stage coordinates and (3) generation of a customized geometry file for high-throughput SCMS. Details on the microMS pipeline has been provided in previous work[2–5], and the software package can be downloaded from https://neuroproteomics.scs.illinois.edu/microMS.htm.

Computational workflow

The key steps of our computational pipeline include (1) tissue MSI and SCMS reconstruction from reduced transients; (2) multimodal image registration and 3D reconstruction; (3) regional analysis; and (4) single-cell and tissue data integration. Detailed protocols to reproduce our results in the manuscript are provided with an example data set shared for learning the protocols. Our computational processing codes are implemented mostly in Python as well as MATLAB (for image registration). Code repository: https://github.com/richardxie1119/MEISTER

Summary of key scripts and notebooks:

`train_signal_model.py`: model training of high-resolution MSI reconstruction
`run_deepmsi.sh`: A bash script for specifying key parameters of the reconstruction and inference.
`embed_3DCoronal.ipynb`: Parametric uniform manifold approximation and projection (UMAP) for low-dimensional embedding of MSI data.
`img_reg_updated.mat`: Affine and B-Spline transformation for image registration
`mri_reg_atlas.ipynb`: Visualization of 3D MSI data of the rat brain.
`regional_analysis.py`: Regional analysis of 3D MSI data of the rat brain.
`tissue_sc_mapping_deepmsi.ipynb`: Integrative tissue and SCMS analysis.

Summary of the minimal datasets to run the pipeline (data can be accessed at https://doi.org/10.13012/B2IDB-9740536_V1, raw transients file needs to be downloaded separately from this link: https://uofi.box.com/s/oagdxhea1wi8tvfij4robj0z0w8wq7j4 and placed into the .d folder within the Bruker data folder):

`saved_model.zip`: trained model weights (decoder and regressor) on high-resolution MSI data used in the manuscript.
`20210930_ShortTransient_S3_5.zip`: serial sections of rat coronal sections acquired with low mass resolution as input for reconstruction in the manuscript.
`coronal3D_propagated_decoded.h5`: reconstructed and processed MSI data of serial sections of rat coronal sections.
`peak_data_sc_regional_decode.imzML`: peak data of 13,566 single rat brain cells.
`coronal3D_slide3_5_R00_propagated_decoded.imzML`: peak data of a representative rat brain coronal section.

Supplemental Protocol 1: Network training and reconstruction

Protocol 1 describes the steps to take the raw MSI data obtained from tissue mostly at low mass/spectral resolution and output enhanced spectra with a high mass resolution. Specifically, this protocol provides instructions for training the proposed autoencoder/regressor network using tissue MSI data (or other hyperspectral data sets, such as single-cell MS data) collected at high resolutions to enable the reconstruction of high-resolution mass spectra from other tissue slices collected at low resolutions. The required data for implementation includes (a) one or more tissue slices imaged at a high spectral resolution (training data) and (b) data of tissue slices collected at a low resolution (high throughput). **Raw transient data** are used and no prior processing is necessary.

Training

1. Install all necessary dependencies and activate the MEISTER environment. Installation details can be found on Github, at https://github.com/richardxie1119/MEISTER
2. Train the autoencoder and regressor using the high-resolution data. Model training is performed via the script `train_signal_model.py` which is executed in the command line using argparse for flexible implementation. An example of implementation is shown below,

```
python train_signal_model.py --train_ROI R00 R01 R02  --path_file
file_dir_coronal_3D_train.json --batch_size 256 --epochs_encoder
10 --epochs_regressor 30 --latent_dim 32
```

   How these parameters are set is explained as follows.

   `R00`, `R01`, and `R02:` correspond to the tissue sections obtained at high-resolution. To obtain these labels, navigate to the Bruker data file (xxx.d) containing the high-resolution data. Open the XML source file. From here, labels can be manually identified as shown (here, using VS Code):

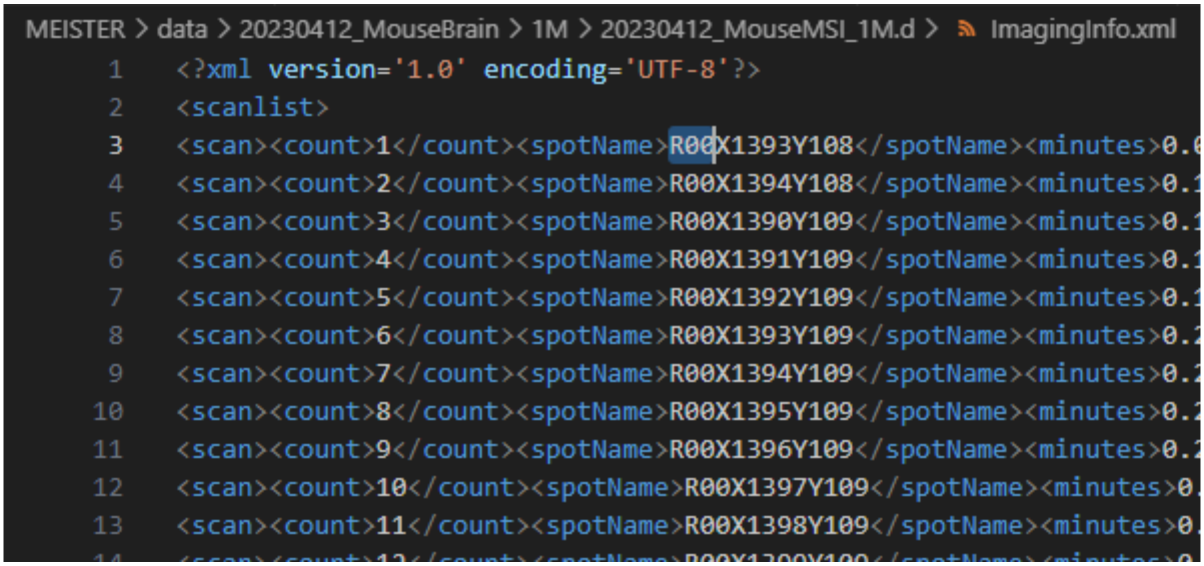

```
MEISTER > data > 20230412_MouseBrain > 1M > 20230412_MouseMSI_1M.d > ImagingInfo.xml
<?xml version='1.0' encoding='UTF-8'?>
<scanlist>
<scan><count>1</count><spotName>R00X1393Y108</spotName><minutes>0.
<scan><count>2</count><spotName>R00X1394Y108</spotName><minutes>0.
<scan><count>3</count><spotName>R00X1390Y109</spotName><minutes>0.
<scan><count>4</count><spotName>R00X1391Y109</spotName><minutes>0.
<scan><count>5</count><spotName>R00X1392Y109</spotName><minutes>0.
<scan><count>6</count><spotName>R00X1393Y109</spotName><minutes>0.
<scan><count>7</count><spotName>R00X1394Y109</spotName><minutes>0.
<scan><count>8</count><spotName>R00X1395Y109</spotName><minutes>0.
<scan><count>9</count><spotName>R00X1396Y109</spotName><minutes>0.
<scan><count>10</count><spotName>R00X1397Y109</spotName><minutes>0.
<scan><count>11</count><spotName>R00X1398Y109</spotName><minutes>0.
```

The XML source file will contain info for every free induction decay (FID) obtained, so be sure to scroll to the bottom of the file to get labels for every image.

3. Two .json files are used to specify the pertinent file paths and parameters of the original data, respectively. In the `path_file`, here titled `file_dir_coronal_3D_train.json` include the locations for the imaging .xml files and the high-resolution data. The imaging data is contained in the XML source code and the same file should be specified for the three paths for training the data. Because the training is only being performed on the high-resolution data at this stage, the XML file for the high-resolution data can be put for all three `imaging_info_path` inputs. The `ser_file_path` contains the raw transients for the data and is contained in a file titled "ser" in the Bruker .d file. Likewise, the same file, corresponding to the high-resolution data, can be put for all `ser_file_path` inputs. The absolute path can be used, or the relative path if the data is contained within the MEISTER folder.


```
{"parameter_file_path": "./data/parameters/parameters_coronal_3D_slide3_5.json",
    "imaging_info_path_BASIS": "./data/20210930_LongTransient_2kHz_S4_S6/20210930_MRI_MSI_LongTransient_S4_S6.d/ImagingInfo.xml",
    "imaging_info_path_HR": "./data/20210930_LongTransient/20210930_Coronal_MSI_LongTransient_S3_S5.d/ImagingInfo.xml",
    "imaging_info_path_LR": "./data/20210930_ShortTransient_S3_5/20210930_ShortTransient_S3_5.d/ImagingInfo.xml",
    "ser_file_path_BASIS": "./data/20210930_LongTransient/20210930_Coronal_MSI_LongTransient_S3_S5.d/ImagingInfo.xml",
    "ser_file_path_HR": "./data/20210930_LongTransient/20210930_Coronal_MSI_LongTransient_S3_S5.d/ser",
    "ser_file_path_LR": "./data/20210930_ShortTransient_S3_5/20210930_ShortTransient_S3_5.d/ser"}
```


4. The second .json files is used to input experimental parameters obtained for the high-resolution data.

MEISTER > {} parameters_coronal_3D_train.json > ...


```
{"project_name": "coronal_latent32_epoch10_25um_run2",
    "pixel_num_HR": 4000,
    "fid_length_HR": 1048576,
     "sw_h": 1428571.4285714286,
      "n_basis": 150,
       "fid_length_LR": 65536,
        "CALIB": [107798997.08328888, 5.694034141537156, 0]}
```


Specify a desired name for the project and input the required parameters. Most importantly, `fid_length_HR` is the number of data points collected per transient for the high-resolution data.

5. Returning to the `train_signal_model.py` command-line script (Step 2), specify the desired batch size (e.g., `--batch_size 256`), number of epochs for the encoder (e.g., `--epochs_encoder 10`) and regressor, and the number of latent dimensions (e.g., `--latent_dim 32`). Note that omitting these parameters will use the default numbers as specified in `train_signal_model.py`. The batch size should be a power of two. The number of epochs can be increased or decreased during optimization if the model is underfit or overfit, respectively.
6. Once all parameters are set, execute the training prompt, shown in (Step 2), in the terminal. Loading data from an SSD and running tensorflow on a GPU is recommended for optimum computational efficiency.
7. When training is complete, two folders containing information for the decoder and regressor will be written to the project folder where the code is stored. By default, the

folders will have the same name as the project_name (by default, MEISTER) with the "_regressor" and "_decoder" suffix.

Reconstruction/Inference

8. Once the autoencoder and regressor are trained, use the regressor to reconstruct the low-resolution data. Reconstruction is implemented with a bash script, with the example script titled `run_deepmsi_3Dcoronal_slide1_2.sh`. Open this file to set the reconstruction parameters.

```bash
#!/bin/bash
export PATH=%PATH:/mnt/c/Users/Richard/Anaconda3/envs/deepmsi

out_dir="./processed_data"
path_files="./file_dir_coronal_3D_slide3_5.json"
decoder_dir="./saved_model/coronal3D_latent32_epoch10_decoder"
regressor_dir="./saved_model/coronal3D_latent32_epoch10_regressor"
recon_ROI=(
    "R00"
)
mz_range="150 1100"
if_process_raw="True"
if_simu='False'
embedding='True'

for ((i=0;i<${#recon_ROI[@]};++i)); do
    printf "Processing data for region %s ...\n" "${recon_ROI[i]}"
    python.exe deep_recon.py  --path_file $path_files --out_dir $out_dir --embedding $embedding --recon_ROI ${recon_ROI[i]}
     --decoder_dir $decoder_dir --regressor_dir $regressor_dir --mz_range $mz_range  --if_process_raw $if_process_raw --if_simu $if_simu
done
```

As in setting up model training, the ROI (here, recon_ROI) can be found in the XML source file. Select the ROIs (e.g., tissue slices) with low-mass-resolution data to be reconstructed.

9. Execute the bash script in the terminal. This will run `deep_recon.py` with the specified parameters.

Reconstruction Output

10. Check the output folder. The following pickle files should be written:
    `encoded_pred.pkl`: the predicted features from the low-resolution data.
    `avgsp_decoded.pkl`: the average mass spectrum from the reconstructed data
    `peak_list_decoded.pkl`: the peak list obtained by performing peak detection on the average mass spectrum.
11. Check the reconstruction results. To quickly look at the reconstruction results load `propagated_decoded.pkl`, which stores the pixel-wise peak lists and the relative pixel coordinates. Now specify the path of the input and output directory. For convenience, define a simple for loop to load the output from multiple tissue sections. Here, `input_dir` is the data folder, and `out_dir` is the folder named `imz` that is generated by the code in the `processed_data` folder. Next, edit the `slice_order` to include the output from each of the slices.

```python
input_dir = 'C:/Projects/MEISTER/processed_data/Run2/'
out_dir = 'C:/Projects/MEISTER/processed_data/imzml/'
slice_order = ['25um_run2_R00','25um_run2_R01','25um_run2_R02','25um_run2_R03','25um_run2_R04','25um_run2_R05','25um_run2_R06',
               '25um_run2_R07','25um_run2_R08','25um_run2_R00','25um_run2_R10','25um_run2_R11']
```

12. The strings specified in `slice_order` will be used to load each of the decoded slices in `peak_list_names`. In the filler code provided, the first file is named `coronal_latent32_epoch10_25um_run2_R00_propagated_decoded` and a total

of 12 slices are included for subsequent 3D stacking. The following code will convert the peak lists pickle files into standard imzML format.

```
intens_mtxs = []
mz_set = []
mz_list = []
peak_list_names = ['coronal_latent32_epoch10_{}_propagated_decoded'.format(slice) for slice in slice_order]

coords= []
names = []
for peak_list_name in peak_list_names:
    coord = pklist2imzML_3D(input_dir,out_dir,peak_list_name)
    coords+=list(coord)
    names+=[peak_list_name]*len(coord)
```

13. To quickly look at just one section and get the intensity matrix (without binning), simply load the data for only one pickle file `slice_order`.

```
mz,peak_list,coord = load_data('./processed_data/Run2/coronal_latent32_epoch10_25um_run2_R00_propagated_decoded.pkl')

intens_mtx = np.array([peak_list[i]['intensity'] for i in peak_list])
intens_mtx = intens_mtx/intens_mtx.mean(1).reshape(-1,1)
```

14. To visualize ion images, index through the columns of `intens_mtx` and use the `IonImg_Show` function to obtain the image reconstruction.

```
#plot image of reconstructed tissue slice at a given m/z
plt.imshow(IonImg_show(intens_mtx[:,827],coord).T,cmap='hot',interpolation='gaussian')
```

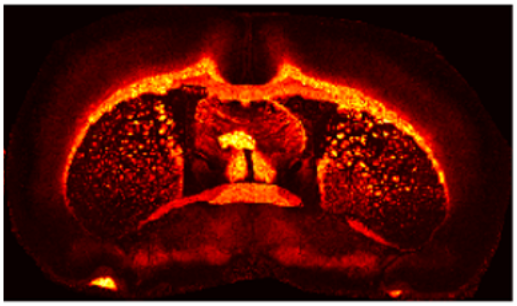

Supplemental Protocol 2: Deformable multimodal image registration using parametric UMAP derived feature maps

This protocol provides detailed information for aligning MSI to a reference atlas, here using a 3D magnetic resonance imaging (MRI) reference. The goal is to correct for tissue deformation and other types of misalignment during the tissue preparation steps. First, pixelwise UMAP is used to obtain the low-dimensional embeddings for the hyperspectral MSI data. The three embeddings with the best morphological contrast, as visually assessed, are combined as RGB channels to form a single grayscale image. An MRI slice that best matches the morphology of the MSI slice was then selected from the MRI atlas.[6] To register the two images, we perform affine registration followed by a B-spline registration. The script provided has functionality to process multiple MSI slices, depending on how many were processed via Supplemental Protocol 1. For simplicity, the case of processing a single MSI slice is shown.

1. The Jupyter Notebook `embed_3DCoronal.ipynb` contains code snippets mentioned below and can be used as a starting point.
2. Run the first cell in `embed_3DCoronal.ipynb` to import necessary packages and dependencies. In the current implementation, functions for performing parametric UMAP are contained in `model.py` and are built on `ParametricUMAP` from `umap.parametric_umap` and facilitated with `tensorflow`.
3. Define the `VAE_model` class which contains the functions necessary for importing data, training, and predicting our model. This class takes the path to the hdf5 file as the input.

```
vae_subspace = model.VAE_model('file_path.h5')
```

4. Next, prepare the training data by

```
train_data = vae_subspace.get_training_data(ratio = 0.1,
group_to_select=vae_subspace.group_names)
```

   -- `ratio`: the fraction of data that is sampled for training. You can choose to train all the groups (i.e., slices) or specify select groups of data for training.
   -- `train_data`: the output as an array with the number of pixels equal to the fraction samples (i.e., 100 if original data has 1000 with ratio = 0.1). The second dimension is the size of the mass spectrum intensity matrix.

5. Train the data by

```
vae_subspace.paraUMAP_train(train_data/train_data.mean(1).reshape
(-1,1),3, False)
```

   Note that the third argument (in this case 3) is the number of latent variables (embedding dimension to use) and can be changed to optimize the model (e.g., for better image contrast).

6. Next, write training information to the original pickle file that will be used during regional analysis. In the next cell, paste in the path to your original .pkl file.

```
with open('file_path.pkl', 'wb') as f:
     pickle.dump(vae_subspace.train_embedding_UMAP, f, protocol =
pickle.HIGHEST_PROTOCOL)
```

7. Optionally, plot the UMAP projections and Cross Entropy (i.e., loss) vs. Epoch to aid in optimizing the model.

8. Predict UMAP embeddings for each slice.

```
vae_subspace.paraUMAP_predict()
```

9. Write and read data to the original pickle file

```
with open ('file_path.pkl', 'wb') as f:
     pickle.dump(vae_subspace.data_info, f, protocol=3)

with open ('file_path.pkl', 'wb') as fp:
     UMAP_datainfo = pickle.load(fp)
```

Here, `vae_subspave.data_info` (and thus, UMAP_datainfo) contains the accurate *m/z*, the indices used, the tic, the coordinates for each pixel, and the embeddings for each slice.

10. Next, visualize the UMAP embeddings. If the data loaded contains more than one slice, specify the name of each slice:

```
groups_show = ['R01']
```

11. Based on the appearance (e.g., contrast) of the obtained embeddings, the parametric UMAP model can be further optimized (e.g., number of latent variables and training data selection). If satisfactory embeddings are obtained, proceed to the notebook **`mri_reg_atlas.ipynb`.** This notebook will be used to further process the data to enable registration to MRI atlas data and perform regional analysis.

12. First, load the pickle file containing the UMAP data. Again, specify the groups/slices to process.

```
with open ('file_path.pkl', 'rb') as fp:
     UMAP_datainfo = pickle.load(fp)

slice_order = ['R01']
```

13. Next, define a function for padding. This is necessary to ensure that the MSI data has the same size as the MRI data.

```
def pad(img, h, w):
    top_pad = np.floor((h - img.shape[0]) / 2).astype(int)
    bottom_pad = np.ceil((h - img.shape[0]) / 2).astype(int)
    right_pad = np.ceil((w - img.shape[1]) / 2).astype(int)
    left_pad = np.floor((w - img.shape[1]) / 2).astype(int)
    return np.copy(np.pad(img, [(top_pad, bottom_pad), (left_pad, 
right_pad)], mode='constant'))
```

14. Next, load the UMAP data. In this step, generate a grayscale image based upon the three embeddings that provide the best contrast (in the example code below, embeddings 1, 7, and 8) as visually ascertained. Then, normalize and center the grayscale image. The variables `umap_img` and `spec_idx_img` will be padded to have the same size as the MRI image that the data will be registered too.

```
import h5py

idx = 0
umap_imgs = []
spec_idx_imgs = []
for slice in slice_order:
    data = UMAP_datainfo[slice_order[idx]]
    coord = data['coordinates']
    embedding = data['embeddings'][:,[1,7,8]]
    umap_img = color.rgb2gray(hyperspectral_vis(embedding,coord,True).transpose(1,0,2))
    umap_img = (umap_img - np.min(umap_img)) / (np.max(umap_img) - np.min(umap_img))
    #umap_img = IonImg(embedding_normal,coord,True,False)
    spec_idx_img = IonImg(np.arange(1,embedding.shape[0]+1),coord,True,False)
    umap_img = pad(umap_img,512,512)
    spec_idx_img = pad(spec_idx_img,512,512)
    umap_imgs.append(umap_img)
    spec_idx_imgs.append(spec_idx_img)
    idx +=1
```

15. Load the MRI atlas and regional labels, e.g., the Waxholm Space Atlas[6] from: https://www.nitrc.org/frs/?group_id=1081

```
import nibabel as nib
file_dir = '../'
epi_labels = nib.load(file_dir+'WHS_SD_rat_atlas_v2.nii.gz')
epi_t2 = nib.load(file_dir+'WHS_SD_rat_T2star_v1.01.nii.gz')
epi_fa = nib.load(file_dir+'WHS_SD_rat_FA_color_v1.01.nii.gz')
```

16. Optional: Visualize the MSI and MRI data.

17. Select an MRI slice that best matches morphologically with the MSI slice to be registered. Fan: you need to repeat these for each slice, correct, which steps are being repeated for each slice. Should specify

    Tim: This can be done for multiple slices simultaneously.. For simplicity, I show the single slice case. I have added a couple lines at the intro paragraph to clarify this point.

```
mri_slice_num = [450]

mri_imgs = []
label_imgs = []
for i in range(len(mri_slice_num)):
    slice_no = mri_slice_num[i]

    label_slice = epi_labels_data[:, slice_no, :].copy()
    mri_slice = epi_t2_data[:, slice_no, :].copy()
    label_slice_mask = label_slice.copy()
    label_slice_mask[label_slice_mask>0] = 1

    mri_slice_masked = mri_slice*label_slice_mask
    #mri_slice_masked = (mri_slice_masked - np.min(mri_slice_masked)) / (np.max(mri_slice_masked) - np.min(mri_slice_masked))
    mri_slice_masked = mri_slice_masked/mri_slice_masked.max()
    #mri_slice_masked[label_slice_mask==0]+=1
    mri_slice_masked = np.flip(mri_slice_masked.T)
    mri_slice_label = np.flip(label_slice.T)

    mri_imgs.append(mri_slice_masked)
    label_imgs.append(mri_slice_label)

    fig,axes = plt.subplots(1,2,figsize=(12,6))
    ax = axes.ravel()
    ax[1].set_title('Waxholm MRI, Slice 450')
    ax[1].axis('off')
    ax[0].axis('off')
    ax[0].set_title('MSI, parametric UMAP, Slice 17, Animal 2')
    ax[1].imshow(np.rot90(mri_slice),'gray')
    #ax[0].imshow(umap_imgs[i],'gray')
    ax[0].imshow(umap_imgsf,'gray')

    plt.show()
```

18. Write the padded MSI and MRI data to our hdf5 file.

```
import h5py

hf = h5py.File('../processed_data/training_5xfad_test_R01_propagated_decoded.h5', 'w')
idx = 0
for slice in slice_order:
    hf.create_dataset(slice+'_umap_img',data=umap_imgs[idx])
    hf.create_dataset(slice+'_spec_idx_img',data=spec_idx_imgs[idx])
    hf.create_dataset(slice+'_mri_img',data=mri_imgs[idx])
    idx+=1
hf.close()
```

19. Open the .mat workbook `img_reg_updated.mat` The next steps in image registration will be performed in MATLAB.

20. Specify the names of the slices to register. As before in the example, only one slice will be registered in the example code.

21. Load in the hdf5 data for both MSI and MRI data.

```
for i=1:length(slice_order)
    mri_img = h5read('../processed_data/training_5xfad_test_R01_propagated_decoded.h5',strcat('/',slice_order{i},'_mri_img'));
    msi_img = h5read('../processed_data/training_5xfad_test_R01_propagated_decoded.h5',strcat('/',slice_order{i},'_umap_img'));
    spec_idx_img = h5read('../processed_data/training_5xfad_test_R01_propagated_decoded.h5',strcat('/',slice_order{i},'_spec_idx_img'));
    mri_imgs(:,:,i) = mri_img;
    msi_imgs(:,:,i) = msi_img;
    spec_idx_imgs(:,:,i) = spec_idx_img';
end
```

22. Specify the moving and target image (“fixed” in the term of this registration package); in this case the MSI data is moving and the MRI is fixed.

```
slice = 1; %change this for every slice
moving = msi_imgs(:,:,slice);
fixed = mri_imgs(:,:,slice);
figure();
imshowpair(moving,fixed,'montage')
%imshow pair creates a composite image showing overlay of two images.
%montage shows A and B next to each other
```

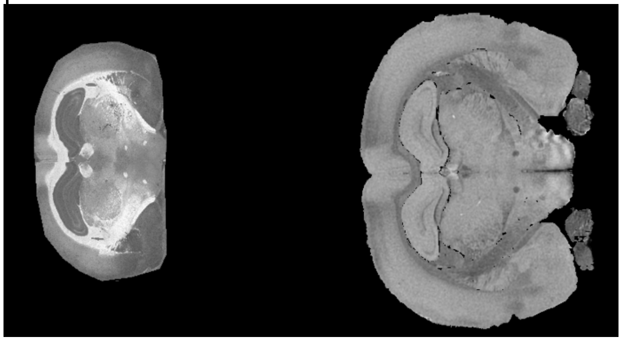

23. Select control points for the transformation. Using about 8 control points is recommended. Choosing points on locations of well-defined contrast and around the edges of the tissue section will give optimum results.

```
%% select control points
cpselect(moving,fixed);
```

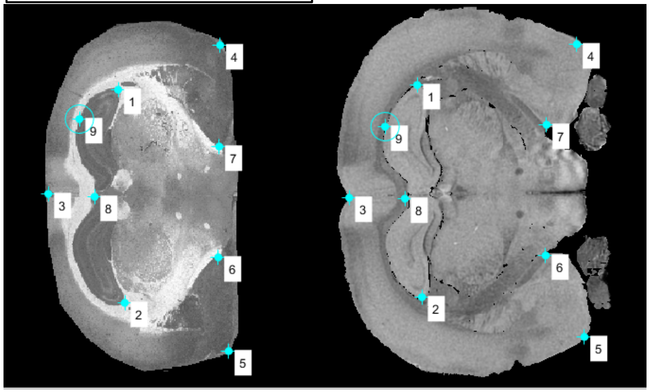

24. Perform coarse registration using affine transformation. A figure will be generated to show the overlay of the MRI and MSI images to verify success of the affine transformation.

```
%%
tformPts = fitgeotrans(movingPoints, fixedPoints, 'affine');
%%
[optimizer,metric] = imregconfig('multimodal');
optimizer.MaximumIterations = 500;
optimizer.InitialRadius = 1e-5;
optimizer.Epsilon = 1e-5;
%%
movingRegisteredAffineWithIC = imregtform(moving,fixed,'affine',optimizer,metric,...
    'InitialTransformation',tformPts,'PyramidLevels',3);
%%
moving_transformed = imwarp(moving, movingRegisteredAffineWithIC,'OutputView',imref2d(size(fixed)));
figure();
imshowpair(moving_transformed,fixed)
```

25. Define parameters for B-Spline registration. These can be optimized depending on the result. However, generally good results have been obtained using the parameters in the

example code below

```
% Main settings
main.similarity='MI';  % similarity measure, e.g. SSD, CC, SAD, RC, CD2, MS, MI
main.subdivide=3;       % use 3 hierarchical levels
main.okno=10;            % mesh window size
main.lambda = 0.05;    % transformation regularization weight, 0 for none
main.single=1;          % show mesh transformation at every iteration

% Optimization settings
optim.maxsteps = 200;   % maximum number of iterations at each hierarchical level
optim.fundif = 1e-5;    % tolerance (stopping criterion)
optim.gamma = 0.1;       % initial optimization step size
optim.anneal=0.8;       % annealing rate on the optimization step
```

26. Run B-Spline registration.
    `[res,newim] = mirt2D_register(fixed,moving_transformed, main, optim)`
    A window will pop up showing the registration and deformation over each iteration. Note that the MIRT package is used for B-Spline and must be included on the MATLAB path. Ensure that convergence is achieved. Note that MSI data with very poor slice quality (e.g., asymmetry between hemispheres) may not be well-amenable to registration.

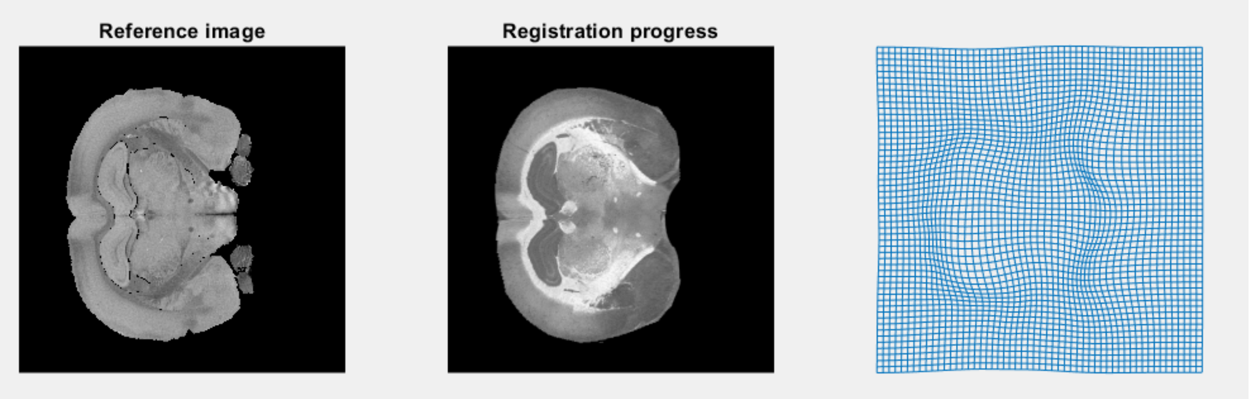

27. Apply the registration to all MSI images and save the .mat data.

28. Return to the python notebook `mri_reg_atlas.ipynb` and load in the .mat data.

```
from scipy.io import loadmat
spec_idx_transformed = loadmat('../image_register/spec_idx_imgs_transformed.mat')['spec_idx_imgs_transformed']
msi_imgs_transformed = loadmat('../image_register/msi_imgs_transformed.mat')['msi_imgs_transformed']
```

29. The remaining code in this notebook can be used to perform regional analysis on the registered data.

Supplemental Protocol 3: MSI data brain regional analysis and MSI+SCMS data integration

This protocol details the procedures for: (1) region-specific analysis for the MEISTER reconstructed 3D MSI dataset post registration (steps 1-8), and (2) integrative analysis of MSI and single-cell mass spectrometry data through our proposed union-of-subspaces fitting (steps 9-14). Python scripts and data used in the publication are provided to run the experiments and free to download online. The codes listed here are relatively concise and only a brief representation of the entire data analysis workflow.

Regional analysis

1. The Python script `regional_analysis.py` and an interactive Jupyter Notebook `regmri_atlas_coronal.ipynb` are provided that contain the codes below as a starting point for users.
2. This is a subsequent step to the multimodal registration step. Processed 3D coronal dataset for this experiment is provided. Following the code `regional_analysis.py` in line 62 to line 91 to load the rat brain MRI atlas files and the corresponding slices.
3. First load both `coronal3D_propagated_decoded_new.h5` and the saved registration transform using the following methods, as shown in line 93 to line 105.

```
file_dir = './processed_data/coronal3D_propagated_decoded_new.h5'
reg = image_registration.Image(data_dir=file_dir,
registration_dir='../image_transform',group_order=slice_order)
reg.load_transform('./image_register/Matlab Registration/
coronal_registration')

reg.load_data(True)
```

4. Next, perform data analysis on individual slices by accessing the intensity matrix through `reg.data[slice_name][‘intensity_mtx’]`, where `reg.data` is a dictionary containing the mass and intensity data for each `slice_name` as defined in Protocol 2.
5. From line 117 to line 137, extract the intensity profiles for each defined brain regions and put them into a single pandas DataFrame object named `region_data_df`
6. In the next step, perform UMAP on the intensity profiles from line 140 to line 158, and the brain regions can be visualized on top of the embeddings. Notice that the random state is fixed. If not, slightly different results will be obtained every time the analysis is

```
region_umap = umap.UMAP(n_components=2,n_neighbors=30,min_dist=0.4,
metric='cosine',random_state=19).fit_transform(region_data)

umap_spec_df = pd.DataFrame({'umap1':region_umap[:,0],
                             'umap2':region_umap[:,1],
                             'regions':region_data_df['region']})
```

run.

7. From line 161 to line 184, use the XGBoost library to train gradient boosted trees to predict brain regions. The one-hot encoded vectors representing brain regions are

passed as the labels as shown in the following code.

```
model = xgboost.XGBClassifier(objective="binary:logistic", max_depth=4,
n_estimators=100, n_jobs=8, verbose_eval=True)

enc = OneHotEncoder(handle_unknown='ignore')
region_onehot = enc.fit_transform(region_data_df['region'].values.reshape(-
1,1)).toarray()
region_encoded = np.argmax(region_onehot,axis=1)

model.fit(X_train, y_train)
```

8. To explain the trained models, the tree explainer is used to provide the feature attributions for each brain region. Mean SHAP values are used as the importance metric for ranking the features. The full code including plotting the feature importance heatmaps and SHAP images is from line 186 to line 242.

```
explainer = shap.TreeExplainer(model)

shap_values = explainer.shap_values(region_data)

mean_shap = np.abs(SHAP_values).mean(1)
```

Single-cell MS to tissue MSI mapping

9. Once regional analysis is complete, move forward with integrating the SCMS data with the MSI data to map their spatial contributions through the union-of-subspaces (UoSS) fitting. The detailed workflow can be found in the notebook `tissue_sc_mapping_deepmsi.ipynb`. First, load the processed single-cell data through the DATSIGMA python libraries[7].

```
from scMSData import scMSData
from analysis import scMSAnalysis

scms_decode = scMSData()
scms_decode.loadimzMLData('../peak_data_sc_regional_decode.imzML')
```

10. Load the tissue MSI data and match the mass features between the two datasets with a mass tolerance of 3 ppm. The `tiss_decode` and `scms_decode` objects contain the intensity matrix with the same number of *m/z* features but rows represent pixels and

cells respectively.

```
intens_array, mz_bins_use, c = extractMZFeatures(tiss_data, ppm=3,
mz_range=(400,1100), feature_n=0.2)

tiss_decode = scMSAnalysis()
tiss_decode.preprocessing(tiss_data,norm_method='rms',feat_drop_rate=0.05,c
ell_drop_rate=0.05)

scms_decode.getIntensMtxData(ppm=3, feature_n=0.05, mz_range=(400,1100),
mz_features=mz_bins_use)

sc_decode = scMSAnalysis(metadata)
sc_decode.preprocessing(scms_decode.intens_mtx,norm_method='rms',feat_drop_
rate=0.05,cell_drop_rate=0.05)
```

11. First, analyze the single-cell data by running the analysis pipelines provided in DATSIGMA libraries[7]. Extract the cluster assignments from the `sc_decode` object.

```
sc_decode.get_labels(['types','batch'])
sc_decode.analyze(n_neighbors=30, n_pcs=40, min_dist=0.5,
                  resolution=0.25,categories=['types','leiden'],log=False)
```

12. For the next step, use nonnegative matrix factorization (NMF) to extract the basis from each cluster of our single-cell data. In this case, for each cluster, extract the top 20 nonnegative basis components from the single-cell intensity profiles.

```
sc_basis = get_sc_basis(sc_decode.intens_mtx,
                        list(sc_decode.metadata['leiden']),k=20)
```

13. To fit the extracted basis to the imaging data, use nonnegative least squares to fit the all basis components and obtain model weights `coef`, corresponding to each individual pixel. The dimension of `coef` is the number of pixels by the number of single-cell basis components.

```
coef,_ = fit_tiss_sc_basis(data = tiss_decode.intens_mtx.values,
                           basis = sc_basis_nmf, positive = True)
```

14. To visualize the fitting results at the tissue level, parse the coefficients for a particular single-cell cluster, take the norm of the coefficients for each pixel, and map it pixel-by-pixel to form a coefficient map for this cluster (e.g., Fig. 5c).

```
img = IonImg_transform(np.linalg.norm(coef[:,i:i+20],axis=1),
                       spec_idx_transform, False)
plt.imshow(img,cmap='viridis', interpolation='gaussian')
```

References

1. Xie, Y. R., Castro, D. C., Rubakhin, S. S., Sweedler, J. V. & Lam, F. Enhancing the Throughput of FT Mass Spectrometry Imaging Using Joint Compressed Sensing and Subspace Modeling. *Anal. Chem.* **94**, 5335–5343 (2022).
2. Comi, T. J., Neumann, E. K., Do, T. D. & Sweedler, J. V. microMS: A Python Platform for Image-Guided Mass Spectrometry Profiling. *J. Am. Soc. Mass Spectrom.* **28**, 1919–1928 (2017).
3. Neumann, E. K., Ellis, J. F., Triplett, A. E., Rubakhin, S. S. & Sweedler, J. V. Lipid Analysis of 30 000 Individual Rodent Cerebellar Cells Using High-Resolution Mass Spectrometry. *Anal. Chem.* **91**, 7871–7878 (2019).
4. Bhaduri, A., Neumann, E. K., Kriegstein, A. R. & Sweedler, J. V. Identification of Lipid Heterogeneity and Diversity in the Developing Human Brain. *JACS Au* **1**, 2261–2270 (2021).
5. Castro, D. C., Xie, Y. R., Rubakhin, S. S., Romanova, E. V. & Sweedler, J. V. Image-guided MALDI mass spectrometry for high-throughput single-organelle characterization. *Nat Methods* **18**, 1233–1238 (2021).
6. Kleven, H. *et al.* Waxholm Space atlas of the rat brain: a 3D atlas supporting data analysis and integration. *Nat Methods* **20**, 1822–1829 (2023).
7. Xie, Y. R. *et al.* Data-Driven and Machine Learning-Based Framework for Image-Guided Single-Cell Mass Spectrometry. *J. Proteome Res.* **22**, 491–500 (2023).

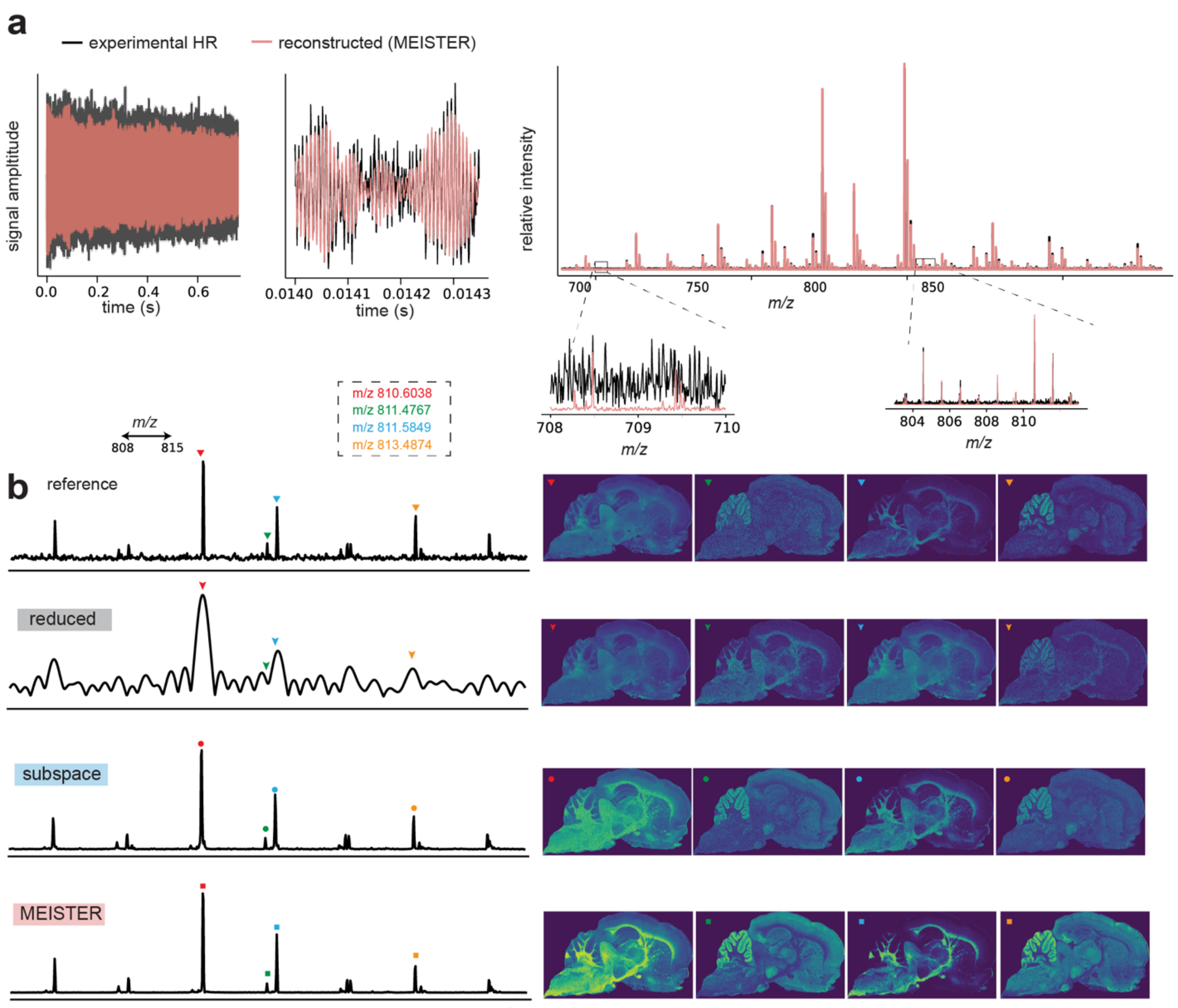

Supplementary Figure 1| a, Experimental and reconstructed transients (1M temporal points for 0.731 s duration) and the transformed mass spectra. **b**, From top to bottom rows: reference, reduced, subspace and MEISTER reconstructed mass spectra (left column), and ion images formed from signals indicated by the colored markers (right column). The data were from a validation data set of a sagittal section adjacent to the training section.

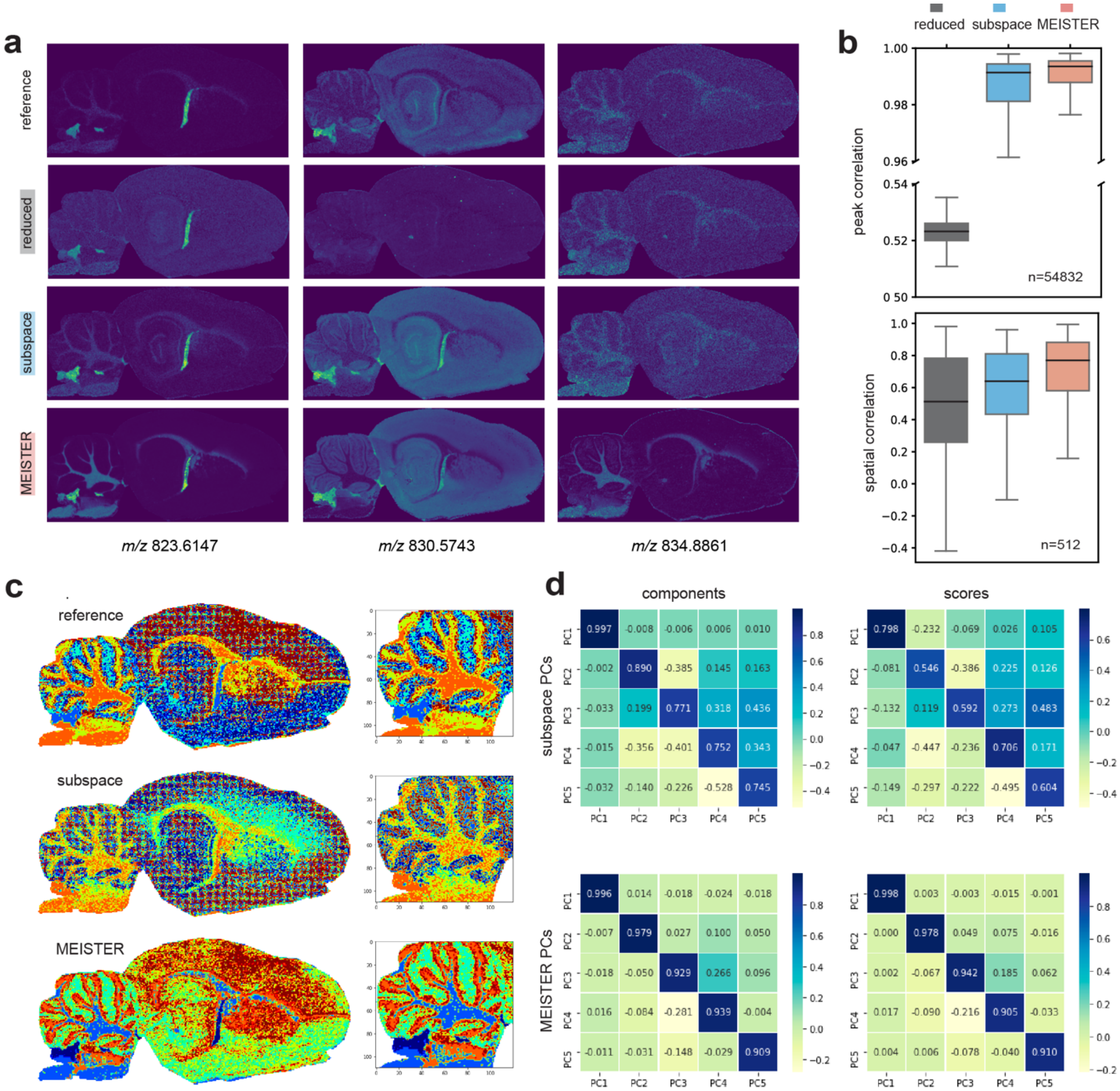

Supplementary Figure 2| a, From top to bottom rows: reference, reduced, subspace, and MEISTER reconstructed ion images from a validation data set acquired on a sagittal section 2 mm away from the training section. **b**, Peak and spatial correlation scores compared to the reference. Data in boxplots are shown as median values (center) with the interquartile range (box), and the whiskers extend to 1.5 times the interquartile range. **c**, K-means clustering assignments for the reduced and reconstructed data by the subspace approach and MEISTER. **d**, Pearson correlation coefficients between top-5 PCs extracted from the experimental reference versus from the data reconstructed by subspace (top) and MEISTER (bottom).

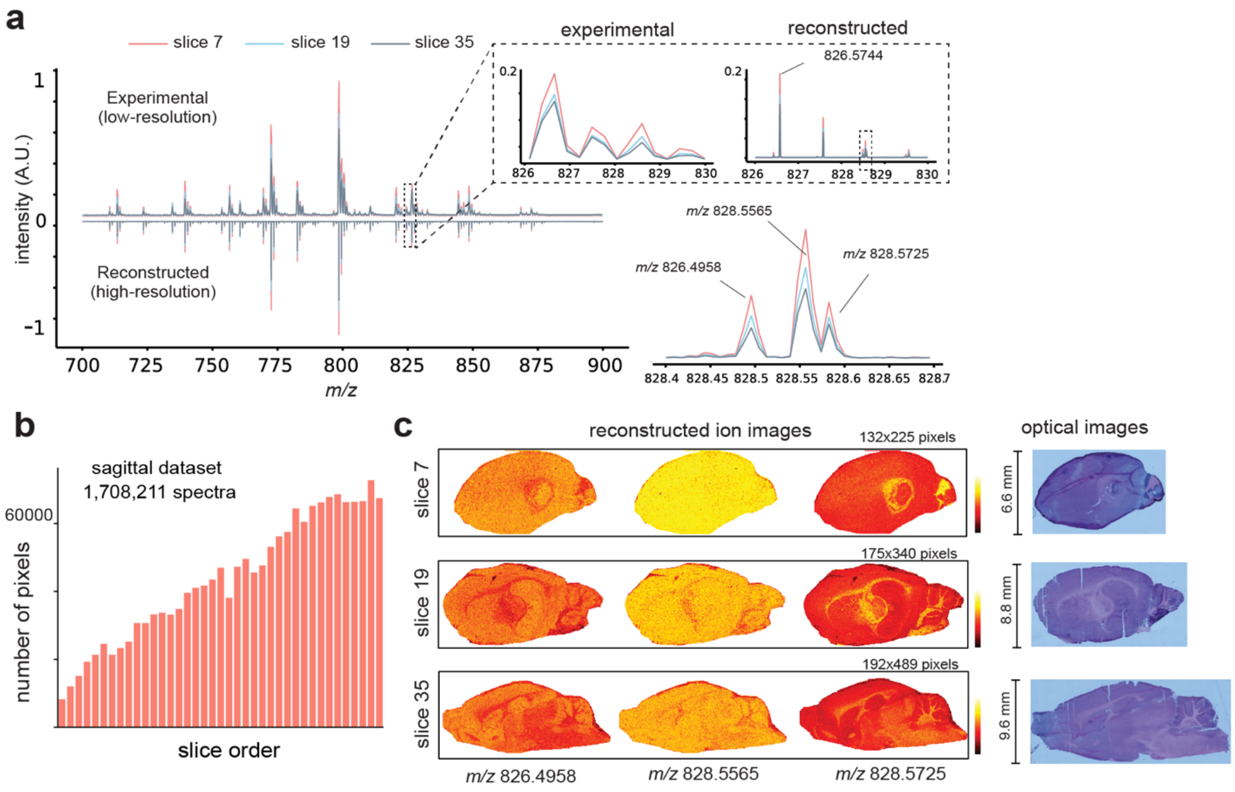

Supplementary Figure 3| Reconstruction of the 3D sagittal data set showing a, Average mass spectral from 3 sagittal sections obtained from MEISTER reconstruction. **b,** Number of pixels versus the slice order for the 3D rat sagittal data set. **c,** Ion images reconstructed by MEISTER (left) and the optical images from the same tissue sections.

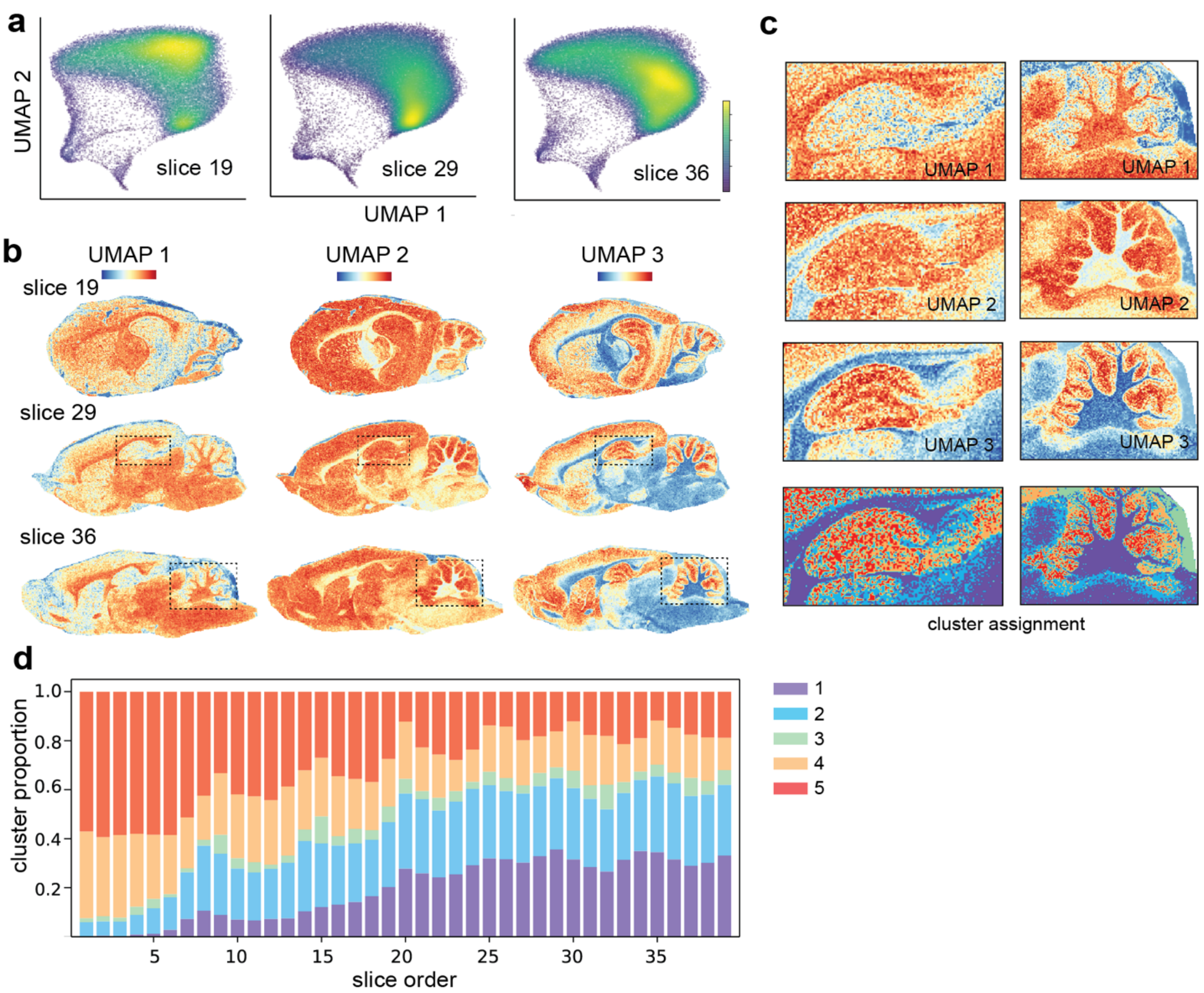

Supplementary Figure 4| a, Parametric UMAP embeddings of the 3D sagittal data set, data shown on 3 representative sections. **b,** Feature images formed by individual UMAP dimensions. **c,** Zoom-in views of the feature images and the k-means clustering assignments, with **d** showing the cluster proportions for 5 clusters varying with the slice order.

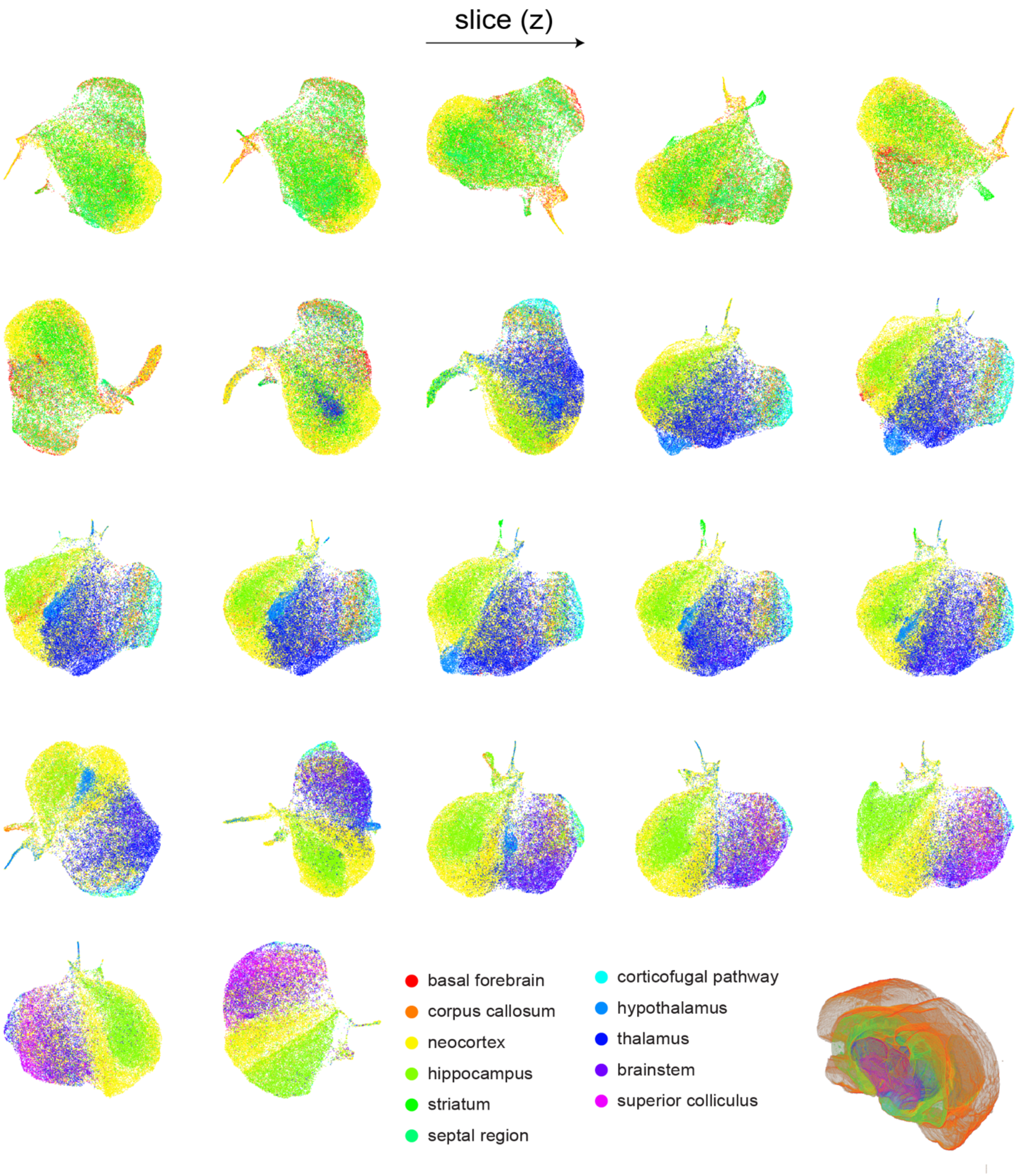

Supplementary Figure 5| UMAP embeddings of representative 2D tissue sections covering all 11 brain regions (colors indicated at the bottom).

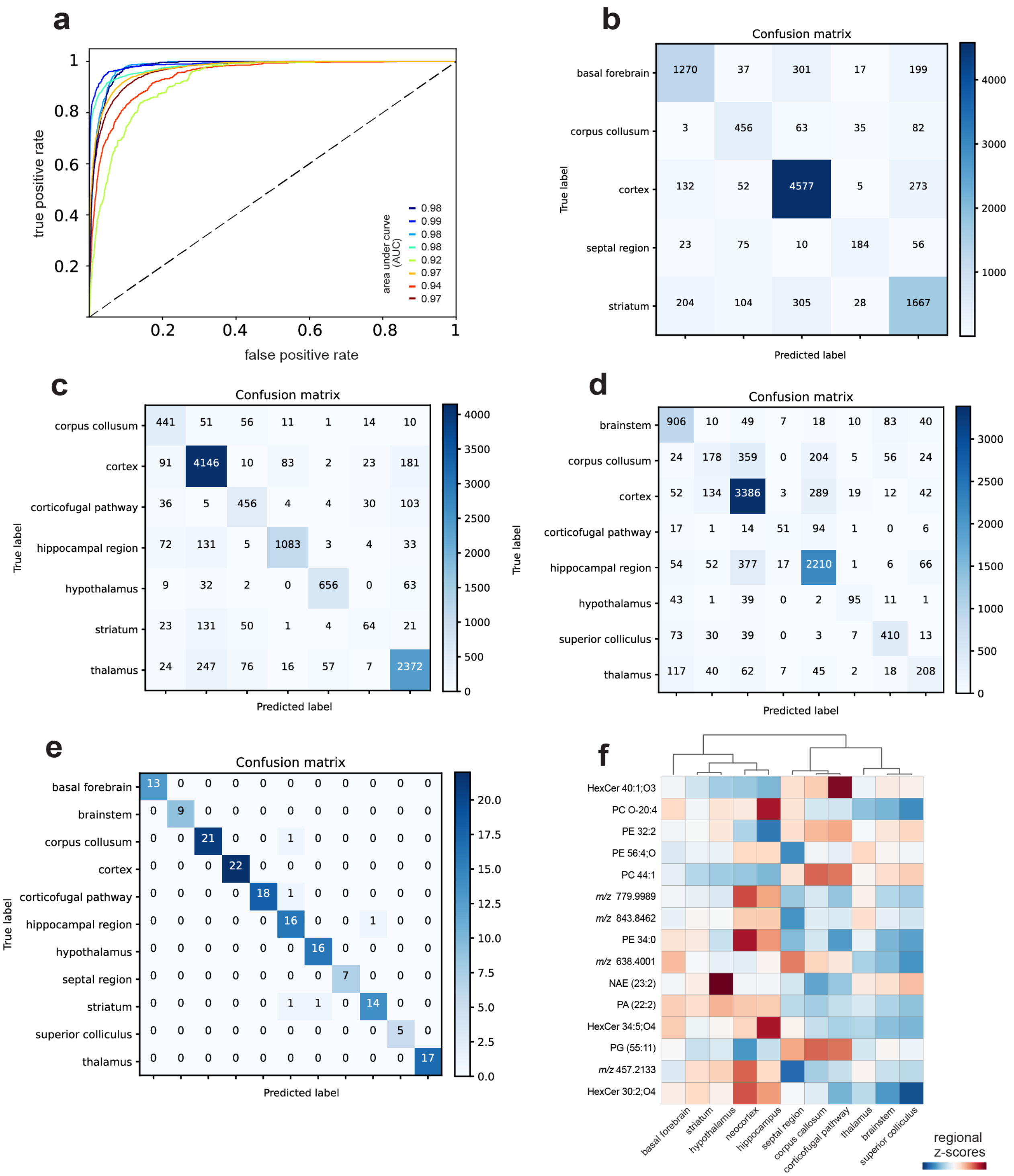

Supplementary Figure 6| Machine learning models trained on 3D MSI pixels to predict brain regions. **a,** The AUC curves of a model trained on a tissue section containing 8 brain regions. **b-d,** Confusion matrices (validated on 20% of pixels) of the models for three tissue sections demonstrated in Figure 4 (top left, top right, and bottom left), and **e,** the confusion matrix of the model trained on the average mass spectra (bottom right). **f**, Mean intensity profiles of the top selected lipid features for different brain structures.

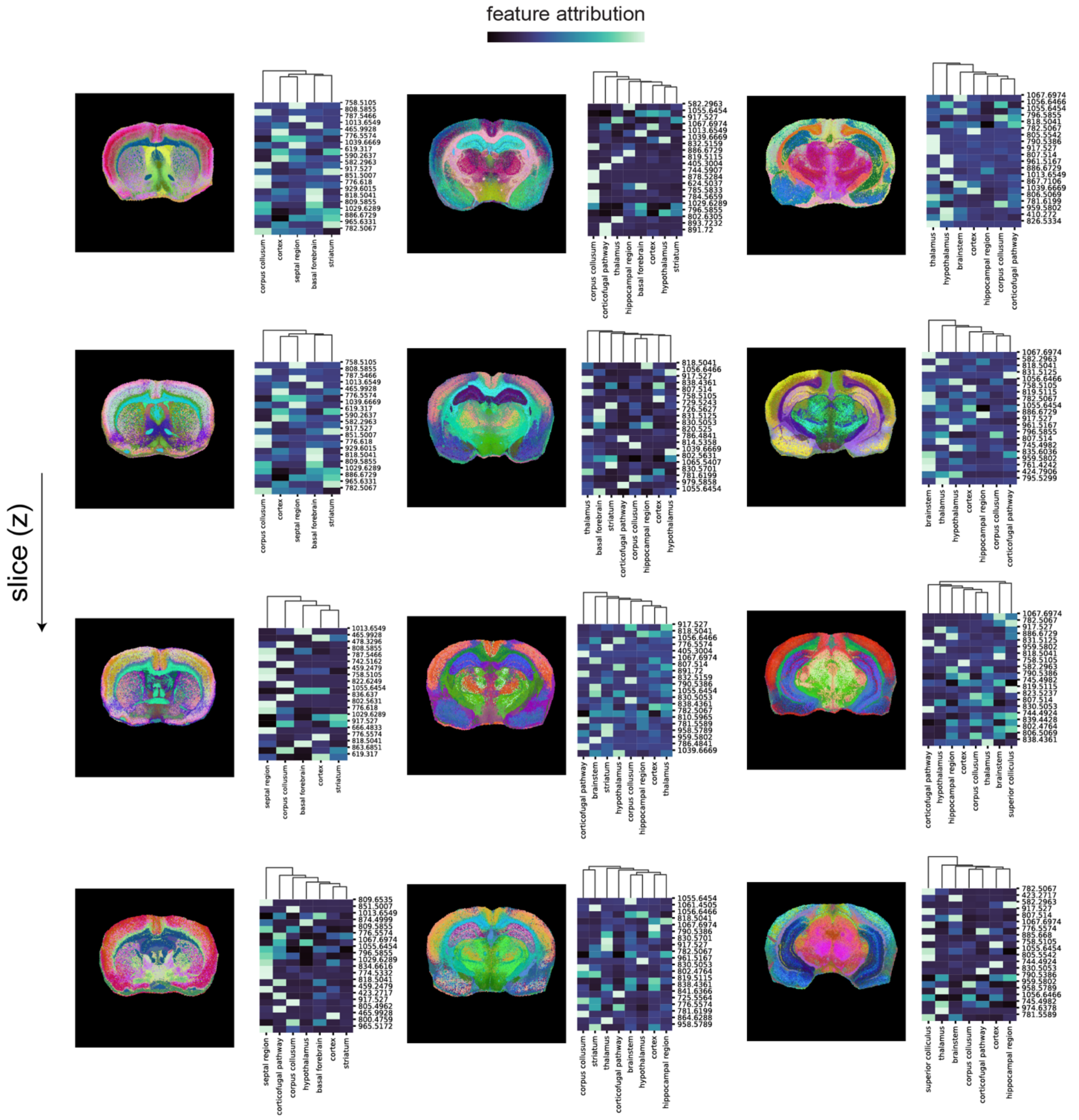

Supplementary Figure 7| Machine learning models trained on individual MSI pixels to predict brain regions and using interpretable machine learning based on SHAP values for top contributing lipid features toward model predictions. For each tissue section, image representations of the UMAP on SHAP values (left) and average SHAP values of top-20 lipid features (right) are shown.

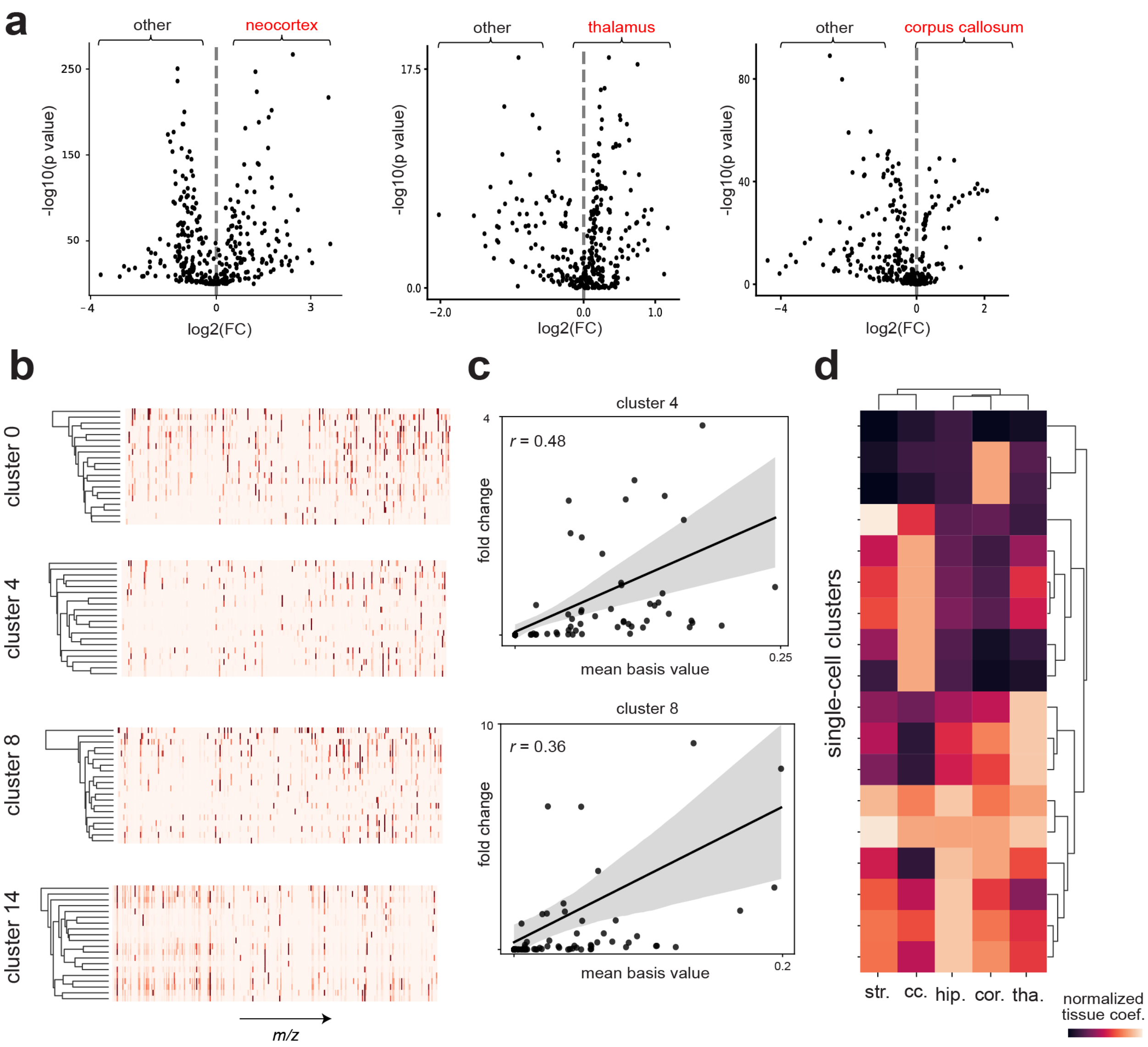

Supplementary Figure 8| a, Volcano plots obtained from differential analysis of regional-specific lipids for 13,566 single cells. p-values were tested by Wilcoxon rank-sum (two-sided) and adjusted by Benjamini-Hochberg procedure. **b**, Additional representative cell-specific chemical dictionaries extracted from clusters. **c**, basis values averaged on 20 dictionary items versus fold change for lipid features in cluster 4 and 8. The shaded band is the 95 percent confidence interval for the linear fit between basis values and fold change. **d**, Analysis of contributions of cell-specific biochemical signatures to brain regions, by summarizing the estimated pixel-wise model weights for each single-cell clusters. Distinct cell compositions can be observed for different regions.

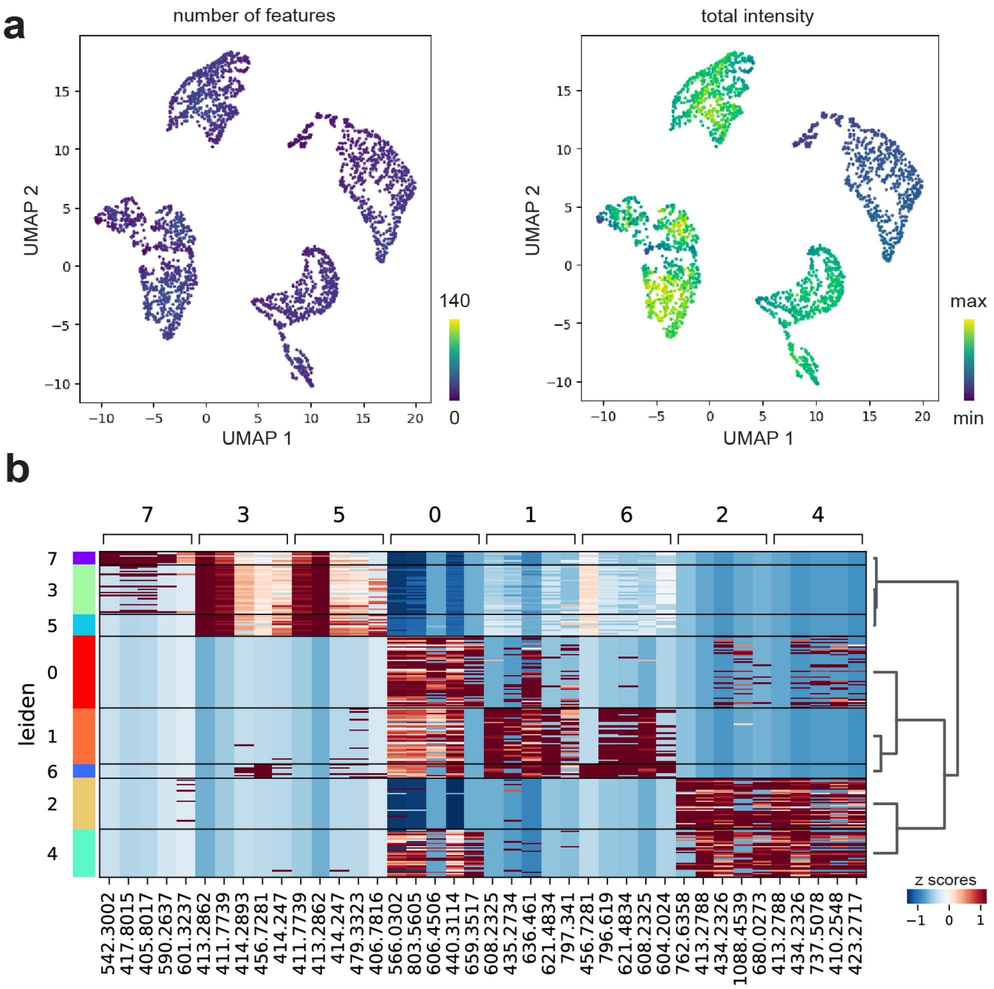

Supplementary Figure 9| a, UMAP of 2,692 hippocampal cells overlaid with number of lipids per cell (left) and total intensity per cell (right). **b**, Heatmap showing the normalized scores (z scores) for the top-5 differential *m/z* features of 8 hippocampal cell clusters.

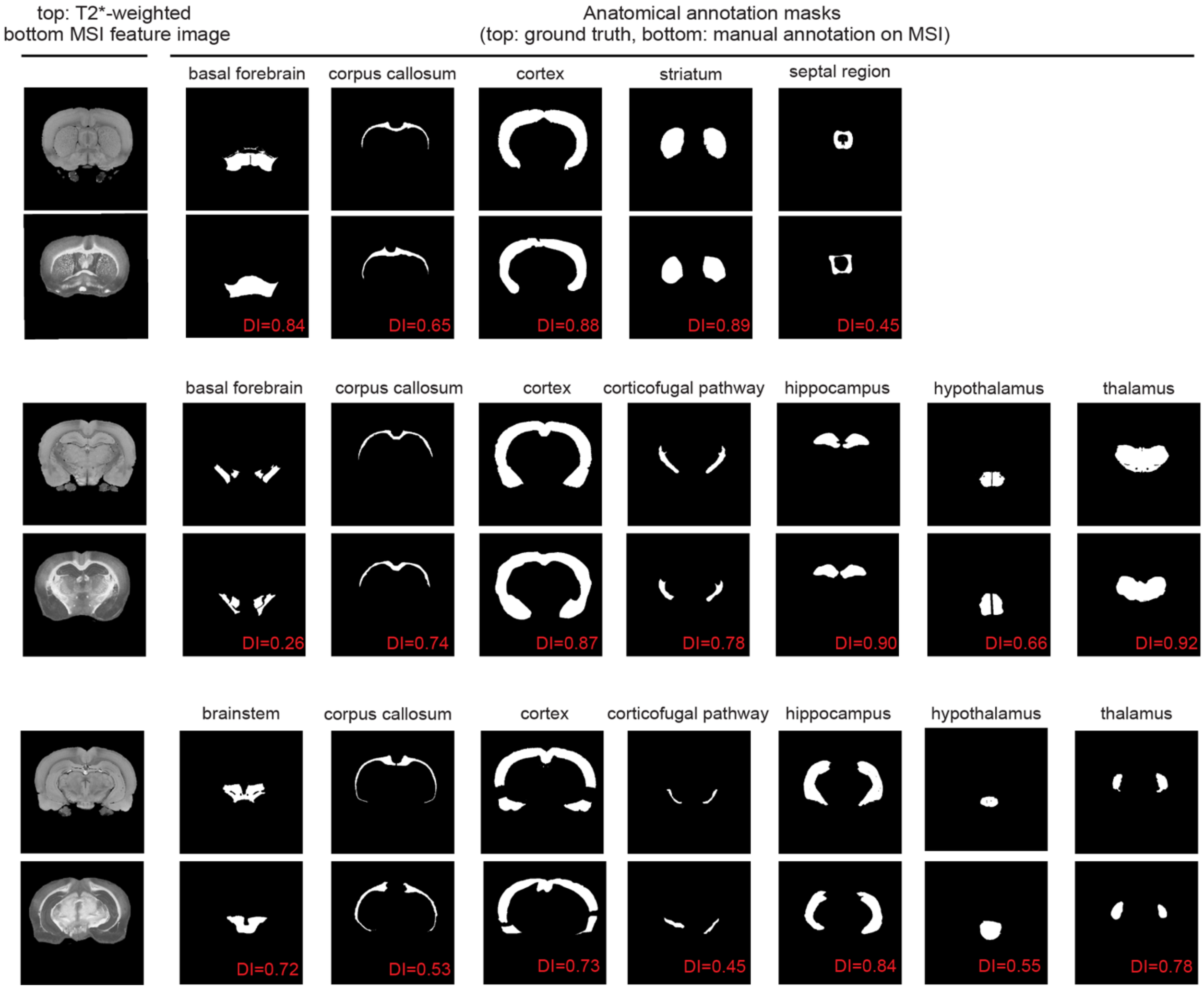

Supplementary Figure 10| Group truth (atlas) and manual annotated (registered MSI) masks for different brain regions. Dice Indices computed for each mask pairs were indicated in red.
